# Prognostic and predictive factors of secondary gliosarcoma: A single-institution series of 18 cases combined with 89 cases from literature

**DOI:** 10.3389/fonc.2022.1026747

**Published:** 2023-01-31

**Authors:** Jinghui Liu, Chen Li, Yuan Wang, Peigang Ji, Shaochun Guo, Yulong Zhai, Na Wang, Meng Xu, Julei Wang, Liang Wang

**Affiliations:** ^1^ Department of Neurosurgery, Tangdu Hospital, The Fourth Military Medical University, Xi’an, China; ^2^ Evidence-Based Social Sciences Research Centre, School of Public Health, Lanzhou University, Lanzhou, China; ^3^ Innovation Center for Advanced Medicine, Tangdu Hospital, The Fourth Military Medical University, Xi’an, China

**Keywords:** secondary gliosarcoma, prognosis, glioblastoma, extracranial metastasis, chemoradiation

## Abstract

**Introduction:**

Secondary gliosarcomas (SGS) are rare malignancies that are diagnosed subsequent to pre-existing glioma. Clinical features and optimal treatment strategies for SGS have not been conclusively established. This study aimed to assess the clinicopathological features and outcomes of SGS.

**Methods:**

We assessed the clinicopathological features and outcomes of SGS via retrospective analysis of data for SGS patients at Tangdu Hospital. Data from SGS patients in prior publications were also analyzed in accordance with PRISMA guidelines.

**Results:**

Eighteen SGS patients who had been treated at Tangdu Hospital between 2013 and 2020 were enrolled in this study. Additional 89 eligible SGS patients were identified from 39 studies. The median age for the patients was 53 years old, and the most common location was the temporal lobe. The most common initial diagnosis was glioblastoma (GBM) (72.0%). Radiology revealed enhanced masses in 94.8% (73/77) of patients. Ten patients (10/107, 9.35%) had extracranial metastases at or after SGS diagnosis. Patients with initial diagnosis of non-GBM and who were younger than 60 years of age were significantly associated with a long duration of disease progression to SGS. After SGS diagnosis, patients with initial non-GBM diagnosis, gross total resection and chemoradiotherapy exhibited prolonged survival outcomes. Patients who had been initially diagnosed with GBM and received both chemoradiotherapy and active therapy after disease progression to SGS, had a significantly longer overall survival than patients who did not.

**Conclusion:**

Initial diagnosis of GBM was a poor prognostic factor for SGS. Patients who underwent gross total resection and chemoradiation had better overall survival outcomes than those who did not. However, during treatment, clinicians should be cognizant of possible extracranial metastases.

## Introduction

Gliosarcomas (GS) are rare malignant central nervous system (CNS) tumors that are characterized by a mixture of gliomatous and sarcomatous elements (1). In the 2016 & 2021 World Health Organization (WHO) classification of tumors of the CNS, GS was classified as a subtype of isocitrate dehydrogenase (IDH)-wildtype GBM (2) and a variant of GBM (3, 4) respectively. Therefore, a similar therapeutic regimen for GS and GBM was recommended by the National Comprehensive Cancer Network (NCCN) (5) and the European Association of Neuro-Oncology (EANO) (6) guidelines. In clinical practice, GS and GBM are also perceived as the same type of lesion and the prognosis of GS patients has been postulated to be comparable to that of GBM patients (7–9). Other studies found that GS has worse prognostic outcomes than GBM (1, 10, 11), with a distinct genomic landscape, indicating that GS are distinctly different tumors from GBM (12).

Among the GBM patients, about 2% are GS cases (1, 13), which are divided into the predominant primary gliosarcomas (PGS) that are *de novo* in origin and secondary gliosarcomas (SGS) that arise from pre-existing gliomas (14–17) and constitute 21% of GS (18, 19). Extremely low incidences of SGS have resulted in a few case reports and studies, creating a paucity of information on its clinical features and optimal treatment strategies. To elucidate on the disease and inform the design of effective treatment strategies for its management, it is important to investigate the prognosis and associated risk factors of SGS.

In this study, data for SGS patients at Tangdu Hospital were retrospectively analyzed, and data for SGS patients in prior published studies were also analyzed. Based on these analyses, we comprehensively elucidate on SGS, specifically its clinical and radiological presentations, pathological diagnosis, and treatment outcomes.

## Methods

### Patient enrollment and data collection

A retrospective analysis was conducted on data from patients treated at Tangdu hospital between 2013 and 2020. The inclusion criteria were: (1) Patients with a history of glioma, (2) Pathological confirmation of GS from subsequent resection. The exclusion criteria were patients with a previously diagnosed intracranial malignant glioma that had GS components. Data from 18 SGS patients were finally analyzed. The ethics committee of Tangdu Hospital approved this study, which had been pre-registered on PROSPERO (Registration number: CRD42022303335).

To obtain patient data from prior studies on GS patients, the following criteria were used: (1) present clinical data of patients, (2) no time restrictions on studies, (3) studies published in English were reviewed by two independent investigators, (4) studies were identified by searching for the terms “Secondary gliosarcoma,” “Recurrence gliosarcoma,” “postirradiation gliosarcoma,” and “post radiotherapy gliosarcoma” alone or in combination in PubMed, EMBASE, Cochrane and Ovid/Medline databases. The reference lists of identified articles were also screened to identify potentially relevant articles.

**Figure 1:**
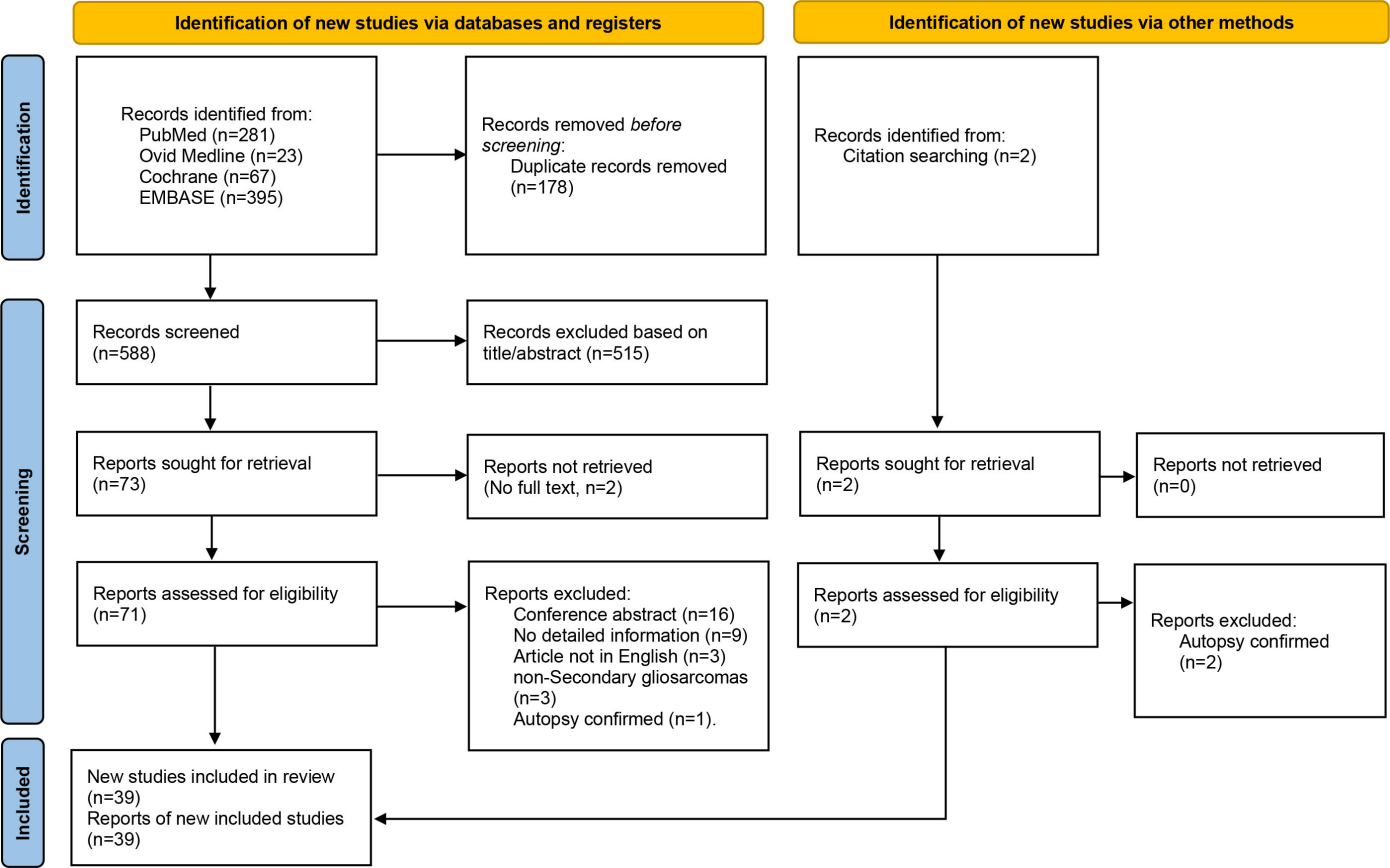
PRISMA flow diagram showing the inclusion and exclusion process for the analysis.

The titles and abstracts of the identified studies were independently screened by two investigators. Studies that did not meet the inclusion criteria were excluded. Then, full articles were screened and those that did not meet the entire inclusion criteria eliminated, leaving 39 studies, from which data on 89 eligible SGS patients were included in the final analysis. These data were reported as per the PRISMA guidelines (**Figure 1**). A total of 107 patients were included in the final analysis. Data that were extracted from patients’ records included: age at diagnosis, sex, tumor location, radiological features of SGS, initial pathological diagnosis, adjuvant therapy for glioma, time from initial diagnosis to SGS, extent of resection for SGS, adjuvant therapy for SGS, survival from SGS, and overall survival after initial diagnosis.

### Quality assessment

To determine the risk of bias in prior studies, two investigators independently assessed the following characteristics: treatment allocation concealment; completeness of outcome data and selective outcome reporting. Disagreements between investigators regarding the risk of bias was resolved by discussion, and when necessary, mediated by a third investigator.

### Statistical analysis

Univariate survival analysis was conducted using the Kaplan Meier method with the logrank test. Factors with p<0.10 on univariate analysis were included in multivariable analyses. Multivariate survival analysis was conducted using the Cox proportional-hazards regression model. Notably, p ≤ 0.05 was set as the threshold for statistical significance. The SPSS^®^ software (Version 20.0) was used for statistical analyses.

## Results

### Demographic characteristics

Clinical records for 18 SGS patients who had diagnosed between 2013 and 2020 at Tangdu Hospital were analyzed. Their clinical information is presented in **Table 1**. Data from these patients were pooled with those from 89 patients in prior SGS studies, totaling to 107 patients. The demographic data for these patients are summarized in **Table 2**. In summary. There were 66 men and 41 women, 93.5% (100/107) of whose records had age data. Median age at SGS diagnosis was 53 years (range 9–82 years). About 72.0% (77/107) of the patients had their radiological data presented, among them, 94.8% (73/77) had enhancing masses. Moreover, 98.1% (105/107) of patients had SGS in known locations; in the temporal lobe (n=51), frontal lobe (n=37), parietal (n=25), and occipital lobe (n=8). Low frequency tumor locations were the insular lobe (n=3), basal ganglia (n=2), and scalp (n=2, 1.9%). In one patient, tumors were located in the cerebellum, brainstem, corpus callosum, dura, subdural, pterygomaxillary region, skull, spinal cord and paranasal sinus. Ten patients had extracranial metastases at or after SGS diagnosis (**Supplementary Table**).

**Table 1:** Clinical data and outcomes of SGS patients in our hospital.

Case	Age	Initial diagnosis	Location of primary glioma	EOR of primary glioma	Adjuvant therapy for primary glioma	Time to SGS (months)	Location of SGS	EOR of SGS	Adjuvant therapy for SGS	OS from SGS (months)	OS from initial diagnosis (months)
n	sex										
1	54-year, F	GBM	temporal	GTR	SRS+TMZ	10.5	temporal	GTR	TMZ	8.5	18.5
2	48-year, M	GBM	temporal	GTR	SRS+TMZ	6.8	temporal	GTR	none	5.3	11.8
3	42-year, F	AO, GBM	frontal	GTR	TMZ	21.3	frontal	GTR	TMZ	7.5	28.8
4	49-year, M	GBM	temporal	GTR	RT+TMZ	14.6	temporal	GTR	none	3.1	17.7
5	41-year, F	GBM	frontal	GTR	RT+TMZ	11	frontal	GTR	Re-op+Bev	16.7	27.7
6	46-year, M	GBM	temporal	GTR	RT+TMZ	13.8	temporal	GTR	TMZ	4.7	18.5
7	49-year, M	GBM	temporal	GTR	SRS	13.6	temporal	GTR	none	3.3	16.9
8	38-year, M	AO	temporal, insular	GTR	none	11.3	temporal, insular	GTR	none	2.1	13.4
9	59-year, F	GBM	frontal, temporal	PR	none	3.9	frontal, temporal	PR	none	1.5	5.4
10	50-year, F	AA	frontal	GTR	RT	19.7	frontal	GTR	TMZ	7.3	27
11	21-year, M	AE	fourth ventricler	GTR	SRS+TMZ	29.1	thoracic, lumbar	GTR	none	2	31
12	67-year, M	GBM	temporal, parietal	GTR	RT+TMZ	19.8	temporal, parietal	GTR	TMZ	25.5	5.7
13	45-year, M	GBM	frontal	GTR	RT+TMZ	12.5	frontal	GTR	none	1.5	14
14	63-year, M	GBM	temporal	GTR	RT+TMZ	14.6	temporal	GTR	none	4.4	19
15	45-year, F	GBM	temporal	GTR	RT+TMZ	40.2	temporal	GTR	TMZ	11.5	51.7*
16	40-year, F	AA	frontal	GTR	RT+TMZ	16	frontal	GTR	TMZ	6.1	22.1
17	27-year, F	LGO	temporal, insular	GTR	None	62.3	temporal	PR	RT+TMZ	16.3	82.6
18	58-year, M	GBM	temporal	GTR	RT+TMZ	62.4	temporo-occipital	GTR	none	2.8	65.2

M, male; F, female; GBM, glioblastoma; AO, anaplastic oligodendroglioma; AA, anaplastic astrocytoma; AE, anaplastic ependymoma; LGO, low grade oligodendroglioma; EOR, extent of resection; GTR, gross total resection; PR, partial resection; SRS, stereotactic radiosurgery; TMZ, temozolomide; RT, radiotherapy; Re-op, reoperation; Bev, bevacizumab; OS, overall survival

*The patient remained alive at the end of follow-up.

**Table 2:** Demographic data for all patients.

Characteristics	n=107
Age, years; median(range)	53 (9-82)
Sex, n (%)	
Male	66 (61.7%)
Female	41 (38.3%)
Tumor location, n (%)	
Temporal	51 (47.7%)
Frontal	37 (34.6%)
Parietal	25 (23.4%)
Occipital	8 (7.5%)
Insular	3 (2.8%)
Basal ganglia	2 (1.9%)
Scalp	2 (1.9%)
Other	10 (9.4%)
Unreported	2 (1.9%)
Extracranial metastases, n (%)	
Yes	10 (9.4%)
No	85 (79.4%)
Unreported	12 (11.2%)
Initial diagnosis, n (%)	
GBM	77 (72.0%)
non-GBM	30 (28.0%)
Adjuvant treatment before SGS diagnosis, n (%)	
Radiotherapy	91 (85.1%)
Chemotherapy	86 (80.4%)
Palliative treatment	9 (8.4%)
Unreported	2 (1.9%)
Surgery of SGS diagnosis, n (%)	
Resection	85 (79.4%)
Biopsy	4 (3.7%)
Unreported	18 (16.8%)
Adjuvant treatment after SGS diagnosis, n (%)	
Radiotherapy	24 (22.4%)
Chemotherapy	57 (54.2%)
Palliative treatment	27 (25.2%)
Unreported	16 (15.2%)

SGS, secondary gliosarcoma.

Most of the patients (82, 76.6%) had prior GBM diagnoses, 77 of which were initial GBM diagnoses. At initial diagnosis, 97 patients were subjected to surgical resection, 4 only received biopsies while 6 patients had unreported treatments. Before SGS diagnosis, 91 and 86 patients had received radiotherapy and chemotherapy, respectively. For chemotherapy, temozolomide (TMZ) was administered to 66 patients. At SGS diagnosis, 85 patients underwent surgical resection, 4 received biopsies only, while 18 had unreported treatments. After SGS diagnosis, 6 patients received radiotherapy only, 40 received chemotherapy only, 18 received chemoradiotherapy, while 16 had unreported treatments (**Supplementary Table**).

### Time to progression to SGS

**Figure 2:**
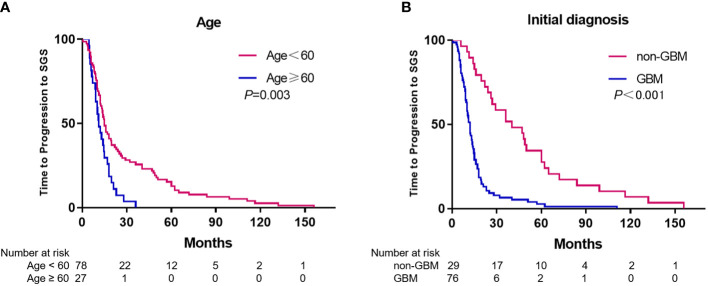
Kaplan-Meier estimates of time to progression to SGS stratified by age **(A)** and initial diagnosis **(B)**.

For 105 patients (98.1%), the median disease progression duration from initial disease diagnosis to SGS was 14.0 months (range 0.5–156 months). Gender and chemotherapy before SGS diagnosis were not significantly associated with duration of disease progression to SGS, as per univariate analysis. Compared with patients younger than 60 years, patients who were aged over 60 years had longer durations of disease progression to SGS (15.0 vs. 11.0 months, p=0.003) (**Figure 2A**). A significantly long duration of disease progression to SGS was seen in patients with initial pathological diagnosis non-GBM, relative to GBM (40.3 vs. 12.0 months, p<0.001) (**Figure 2B**). Furthermore, multivariate analysis revealed that patients with initial diagnosis of non-GBM had significantly longer duration of disease progression to SGS (HR 3.651, 95%CI: 2.269-5.876, p<0.001).

### Survival outcomes post SGS diagnosis

**Figure 3:**
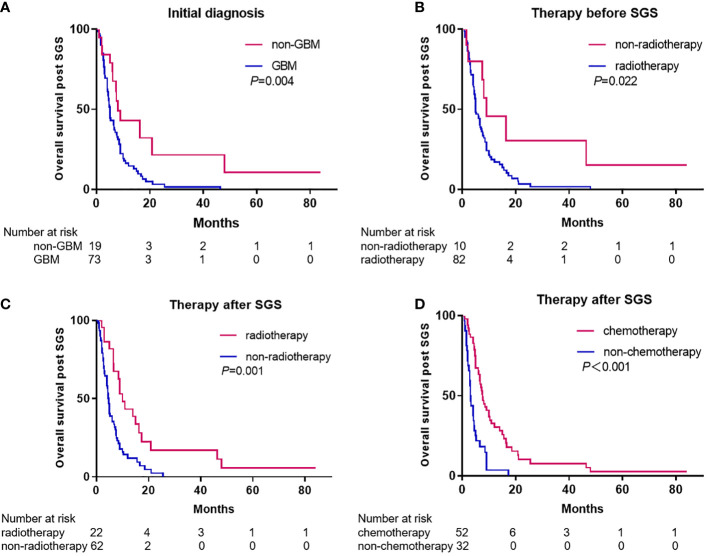
Kaplan-Meier estimates of overall survival post SGS diagnosis stratified by initial diagnosis **(A)**, therapy before SGS **(B)**, and therapy after SGS **(C, D)**.

For 92 patients (86.0%), survival duration post SGS diagnosis was known and had a median of 6.0 months (95% CI, 4.72-7.28). Univariate analysis revealed that post SGS diagnosis, gender, age <60 years and chemotherapy before SGS diagnosis were not significantly associated with survival duration. A significantly longer survival duration post SGS diagnosis was observed in patients with initial diagnoses of non-GBM, compared to GBM (8.0 vs. 5.0 months, *p*=0.004) (**Figure 3A**). Compared to patients who had not been subjected to radiotherapy before SGS diagnosis, we observed a significantly worse survival duration for patients with radiotherapy before SGS diagnosis (7.5 vs. 5.0 months, *p*=0.022) (**Figure 3B**). To analyze the effects of resection of SGS, only data for patients from Tangdu Hospital were used, as that from prior studies often lacked the resection extent. After SGS diagnosis all patients underwent surgical resection and gross total resection (GTR) was achieved in 16 (88.9%) of the patients. Compared to subtotal resection (STR), GTR had a significantly longer median overall survival (OS) time (5.3 vs 1.5 months, *p*=0.003).

For patients who received radiotherapy after SGS diagnosis, their survival duration was longer than that of patients that were not subjected to radiotherapy after SGS (10.0 vs. 4.6 months, *p*=0.001) (**Figure 3C**). A longer survival duration was also observed in patients who received chemotherapy after SGS diagnosis, compared to those who did not receive chemotherapy after SGS (7.6 vs. 3.0 months, *p*<0.001) (**Figure 3D**). Compared to patients who received chemotherapy or radiotherapy alone, those who received chemoradiotherapy had longer survival durations (14.0 vs. 6.7 months, *p*=0.006). Notably, among patients with extracranial metastases, the median survival duration from diagnosis of metastasis to death was 3 months (range 1–8 months). Multivariate analysis revealed that either chemotherapy (HR 3.282, 95%CI: 1.987-5.420, *p*<0.001) or radiotherapy (HR 2.737, 95%CI: 1.562-4.796, *p*<0.001) after SGS diagnosis were independent prognostic factors for survival outcomes.

### Survival outcomes of patients with initial GBM diagnosis

For 72 patients (93.5%), the median OS time for patients with initial GBM diagnosis was known and had a median of 18.5 months (range 5.4–65.2 months). For treatment, 67 patients (88.3%) received radiotherapy and chemotherapy. The median survival time post SGS diagnosis was known for 73 patients and had a median of 5.0 months (range 0.73–46.4 months). After SGS diagnosis, 46 patients received adjuvant radiotherapy and/or chemotherapy while seven patients were re-operated on due to SGS recurrence. Compared with patients who did not receive any treatment after SGS diagnosis, patients who treated with adjuvant radiotherapy, chemotherapy and/or re-operated had longer survival outcomes after SGS diagnosis (6.7 vs 2.8 months, p<0.001). Patients who had received radiotherapy and chemotherapy for GBM and active therapy for SGS had a median survival time of 18.6 months.

## Discussion

Gliosarcoma is a rare tumor that is classified as either primary or secondary gliosarcoma. In a recent meta-analysis, incidences of IDH1/2 mutation, EGFR mutation, and MGMT methylation between PGS and SGS were found to be comparable, however, survival analysis revealed that compared with PGS, SGS is associated with significantly worse PFS and OS outcomes (20). A retrospective study from the MD Anderson Cancer Center showed that the median OS outcome from pathological diagnosis of primary and secondary GS were 17.3 months and 10.2 months, respectively (p < 0.01) (21). A retrospective analysis found that PGS patients had significantly high PFS (p < 0.03) and OS (p < 0.031), compared to SGS patients (9). To gain a better understanding of SGS and design effective treatment strategies for its management, apart from our cases, we performed a systematic review and analysis of literature.

To the best of our knowledge, with a total of 107 patients, this is the largest SGS study. Analysis of patient data revealed disease characteristics and optimal treatment strategies. Lesions were most often located in the temporal lobe (48.6%), and GBM was the most common initial diagnosis (72.0%). After SGS diagnosis, aggressive radiotherapy and chemotherapy were most effective therapeutic options.

Clinically, SGS have been defined in different ways, one of which is tumors diagnosed at recurrence after initial GBM diagnosis (14). Another is tumors detected after a high-grade glioma was either resected or irradiated (16, 22, 23). Other studies defined SGS as those arising from non-irradiated WHO grade II glioma (15, 24). Gliosarcoma originating from grade II oligodendroglioma that had been pretreated with radiotherapy has also been reported in other studies (17, 25). Based on the above studies, we propose the definition of SGS as tumors that originate from a pre-existing glioma, usually after radiation treatment.

Extracranial metastasis of CNS tumors is rare due to the blood-brain barrier and the absence of lymphatic vessels in the CNS (26, 27). The reported incidences of extracranial metastases for GBM vary between 0.4–0.5% (26), which is comparatively low than the 11% frequency for GS, which is commonly known to metastasize to the lungs, liver, and lymph nodes (28). In 2010, Han et al. (14) reported 30 cases of confirmed SGS, of which one patient had scalp/subgaleal metastasis. In 2013, 44 SGS cases were reported, of which five patients had extra-cranial metastases (29). In this study, ten patients developed extracranial metastases at or after SGS diagnosis with the lungs being the most common metastatic site (three patients). This implies that SGS is likely to undergo extracranial metastasis, therefore, identification of potential extracranial diseases, during initial diagnosis and continued surveillance is necessary.

The association between extracranial metastases of glioma and prognosis has been previously investigated. In a meta-analysis of 88 cases of extracranial glioblastoma (five were GS) (26), the median time from diagnosis of primary glioblastoma to detection of extracranial metastasis was 8.5 months, while from metastasis to death was 1.5 months, with lung metastasis patients having the worst survival outcomes. Sun et al. (30) reported cases of two patients who developed extracranial metastases after surgery for primary glioma, and died within 2 months of metastasis diagnoses. In this study, the median survival time from diagnosis of metastasis to death was 3 months (range 1–8 months). Therefore, when patients present with dyspnea or physical pain without deterioration of their neurologic status, clinicians should be cognizant of the possibility of metastatic disease.

In this study, among non-GBM patients at initial diagnosis, median durations from initial diagnosis to SGS and median OS post SGS diagnosis were 36 months and 8 months, respectively. These survival durations were comparable to those of patients with secondary glioblastoma (sGBM), which have been reported to be 158.9 weeks (31), and 7.8 months (32), respectively. In contrast, patients with initial GBM diagnosis had significantly shorter survival outcomes as the median duration from initial diagnosis to SGS and median OS post SGS diagnosis was 12.0 and 5.0 months, respectively. This was comparable to that of recurrent glioblastoma (rGBM) patients, who had a median survival time of approximately 6 months (33, 34). This disparity indicates that different initial diagnoses have potentially different clinical and molecular characteristics, such as sensitivity to treatment and IDH mutation rates.

In this study, the extent of resection was a significant prognostic factor for GS and this corroborated extent of resection as a crucial prognostic factor for primary GBM (35, 36), rGBM (37, 38) and sGBM (31, 39). Smith et al. (23) analyzed 22 PGS patients and showed that the extent of resection was a significant prognostic factor in univariate but not in multivariate analysis. Moreover, for 34 GS patients (24 PGS and 10 SGS), those who had GTR at the time of first diagnosis lived longer than those with STR (40), however, this study did not analyze the SGS separately. In tandem with previous studies, we found that the median OS was significantly longer in GTR patients than STR patients, demonstrating that GTR can significantly prolong the OS outcomes of SGS patients.

In this study, for patients whose initial diagnoses were non-GBM and treated with radiotherapy, the median survival time after SGS diagnosis was longer, compared to those treated with only chemotherapy or palliative care (20.9 vs 7.3 vs 2.0 months p<0.001). However, all SGS cases were recurrent gliomas, and thus, some patients were ineligible for re-irradiation. Prior studies have noted the importance of active treatment on survival time of sGBM patients. In a single-center retrospective study of 39 sGBM patients, patients who had been subjected to adjuvant treatment exhibited longer OS, compared to patients without adjuvant treatment (18.3 vs 8.8 months, *p*=0.003) (39). Moreover, Gessler F et al. (31) conducted a retrospective study of 45 sGBM patients and found that radiotherapy and chemotherapy are associated with prolonged OS. Further, patients treated with chemoradiotherapy had significantly longer survival outcomes, compared with those treated with a standalone treatment (87.3 vs 54.3 weeks, *p*<0.001). These results are in tandem with our findings.

A retrospective study from the MD Anderson Cancer Center showed that the median OS time for PGS was 17.5 months (40), which was similar to that of GBM. Conversely, a multi-center study conducted by Castelli J et al. (41) found that the median OS time for PGS was only 13 months and TMZ chemotherapy was not associated with improved OS, compared to patients who received radiation therapy only. Another study involving 30 SGS patients, who relapsed after progression to GBM, found that the median OS after original GBM diagnosis was 12.6 months (14). Therefore, GS patients tend to have poor prognostic outcomes. However, a study involving 10 SGS patients (9 patients with initial GBM diagnoses and one with anaplastic oligodendroglioma) showed the median OS post original diagnosis as 18.6 months (23). This corroborates our results where the median OS was 18.6 months in patients who had received chemoradiotherapy and active therapy for treatment GBM and SGS respectively. Compared with previous studies, we enrolled a large number of SGS patients, which increased the degree of accuracy and robustness. We show that recurrence of GBM as SGS does not affect the OS time.

The O6-methylguanine DNA methyltransferase (MGMT) promoter methylation is the most important prognostic factor in GBM, especially in relation to temozolomide efficacy (42). The MGMT promoter methylation is also a significant prognostic factor for temozolomide rechallenge in rGBM (43).The MGMT status is significantly associated with OS in temozolomide-treated PGS patients, yet the frequency of MGMT promoter methylation is significantly low in PGS (26.1%) than GBM (54.6%) (8). Furthermore, the median OS time for GS patients with MGMT promoter methylation is 16.4 months versus 9.4 months for those with unmethylated MGMT promoter (44). Singh et al. detected MGMT promoter methylation in five of 16 GS patients who had been treated with temozolomide, however, the MGMT status did not significantly affect OS. Singh et al. (45) detected MGMT promoter methylation in five of 16 GS patients who had been treated with temozolomide, however, the MGMT status did not significantly affect OS. There are no relevant studies on the association between MGMT promoter methylation and OS of SGS patients. Despite this study having 16 patients with known MGMT status, no further statistical analyses were performed as only four and seven of the sixteen patients had MGMT methylation and treatment with temozolomide post SGS diagnosis, respectively. Further studies should investigate whether TMZ rechallenge is a treatment option for SGS, especially for those with MGMT promoter methylation.

## Limitation

Although our findings are generally encouraging, this study has some limitations. First, this was a retrospective study, which has its inherent limitations. Second, given that most cases were based on previously published articles, it was inevitable that some clinical data were not available in all studies, such as pre- and postoperative KPS scores, extent of resection and number of chemotherapy cycles. Third, for data from studies that spanned long durations, treatment regimens often differed between patients and treatment-related adverse effects were not always recorded. Fourth, several important molecular markers, such as IDH and telomerase reverse transcriptase (TERT) promoter mutations as well as epidermal growth factor receptor (EGFR) amplification were not available. Studies should aim at elucidating the clinicopathologic features, treatment strategies, and outcomes of SGS patients.

## Conclusion

Despite the rarity of SGS, 107 SGS patients were included in the final analysis, making this the largest study of SGS patients to date. Patients with an initial non-GBM diagnosis had favorable prognostic outcomes. After SGS diagnosis, there was a high risk of extracranial metastasis, and the lung was the most common metastatic site. Extracranial metastases were associated with poor prognoses. Patients with GTR and chemoradiation after SGS diagnosis exhibited better overall survival outcomes, therefore, we recommend that the most suitable SGS treatment strategy is maximal safe resection combined with adjuvant chemoradiotherapy. However, during treatment, clinicians should be cognizant of possible extracranial metastases.

## Data availability statement

The original contributions presented in the study are included in the article/**Supplementary Material**. Further inquiries can be directed to the corresponding authors.

## Author contributions

JL performed the data analyses and drafted the manuscript. CL performed the clinical analyses (imaging data) with YW and contributed to writing of the manuscript. PJ, SG and YZ contributed to data analyses. NW and MX performed the clinical follow up. LW performed the clinical analyses and designed the study with JW. All authors contributed to the article and approved the submitted version.
