# Supplementary material for: Prognostic and predictive factors of secondary gliosarcoma: A single-institution series of 18 cases combined with 89 cases from literature
Source: Front Oncol. 2023 Jan 31;12:1026747. doi: 10.3389/fonc.2022.1026747 (PMC9927223; doi:10.3389/fonc.2022.1026747)
Supplement: Supplementary file 1 [file Table_1.docx]

**Supplementary table: Summary all the cases of SGS**

| Study | Case | Age (yrs) | Initial diagnosis | EOR of primary glioma | Adjuvant therapy for primary glioma | Time to SGS (months) | Location of SGS | OS from SGS (months) | Therapy for SGS | Extracranial metastasis |
| --- | --- | --- | --- | --- | --- | --- | --- | --- | --- | --- |
|  | n | sex |  |  |  |  |  |  |  |  |
| 2000(1) | 1 | 41, F | AA, GBM | Biopsy | NR | NR | B | NR | NR | No |
|  | 2 | 56, F | GBM | Biopsy | NR | 4.0 | P/O | NR | NR | No |
| 2001(2) | 3 | 48, M | GBM | GTR | Rt+Chemo# | 15.0 | F | 9.0 | NR | No |
| 2004(3) | 4 | 48, F | AE | NR | Rt+Chemo# | 22.0 | F | NR | Chemo# | No |
| 2006(4) | 5 | 8, M | GBM | GTR | Rt+Chemo# | 111 | F | 1.0* | NR | No |
| 2007(5) | 6 | 48, F | LGO | STR | PCV | 99 | F | 9.0 | Rt | No |
|  | 7 | 47, M | AO | GTR | Rt+PCV+TMZ | 49 | F | 11* | CIS+DOX | No |
|  | 8 | 35, F | LGO | NR | none | 60 | F/T | NR | NR | No |
|  | 9 | 29, F | AO | STR | Rt+melphalan | 84 | F/Sinus | NR | none | Yes |
|  | 10 | 68, M | LGA | PR | Rt | 6 | T | 5.0 | none | No |
|  | 11 | 44, F | LGO | NR | none | 24 | F/T | 8.0 | NR | No |
| 2008(6) | 12 | 8, M | PA | STR | Rt | 156 | T/I | 48.0 | SR+Rt+TMZ | No |
| 2009(7) | 13 | 28, M | PA | STR | Rt+TMZ | 14.0 | T | NR | NR | No |
| 2009(8) | 14 | 29, F | LGA | GTR | Rt+Chemo# | 116.4 | Cb | NR | NR | No |
| 2010(9) | 15 | 52, M | GBM | STR | Rt+TMZ | 19.0 | F, scalp | 2.0 | SR | Yes |
| 2010(10) | 16 | 26, M | AA | GTR | Rt+Chemo# | 27.0 | F | 12.0* | NR | No |
| 2010(11) | 17 | 48, M | GBM | STR | Rt +HU+CBP+TAM | 5.0 | P/O | 0.73 | none | No |
|  | 18 | 53, M | GBM | STR | Rt+TMZ+BCNU+TAM | 5.5 | F/T/P | 1.6 | none | No |
|  | 19 | 62, M | GBM | GTR | Rt | 4.5 | P | 2.9 | none | No |
|  | 20 | 71, M | GBM | GTR | Rt+TMZ | 7.0 | F | 2.5 | CCNU+TAM | No |
|  | 21 | 56, M | GBM | GTR | Rt | 3.5 | T | 6.5 | ^125^I | No |
|  | 22 | 53, F | GBM | GTR | Rt | 6.0 | T | 4.0 | TMZ | No |
|  | 23 | 54, M | GBM | GTR | Rt+TMZ+E+ IL-13 | 6.0 | T | 4.2 | none | No |
|  | 24 | 50, F | GBM | GTR | Rt+TMZ+E+CCNU+  CBP+Bev | 6.0 | F | 4.4 | heat shock protein | No |
|  | 25 | 58, M | GBM | STR | Rt+TMZ+E | 7.5 | T | 3.0 | SRS | No |
|  | 26 | 46, F | GBM | GTR | Rt+TMZ+Bev | 8.0 | scalp/subgalea | 3.0 | Rt | Yes |
|  | 27 | 50, M | GBM | GTR | Rt+TMZ | 8.5 | F/P | 2.6 | E | No |
|  | 28 | 42, F | GBM | STR | Rt+6-dimethyl protocol | 9.5 | T | 1.7 | none | No |
|  | 29 | 70, M | GBM | GTR | Rt+TMZ | 5.0 | P | 7.6 | IL-13 | No |
|  | 30 | 61, M | GBM | GTR | Rt+SU-101+BCNU | 8.8 | O | 4.0 | TMZ | No |
|  | 31 | 63, M | GBM | STR | Rt+TMZ+Bev+Paz | 10.0 | NR | 4.5 | Bev+IRN | No |
|  | 32 | 46, F | GBM | GTR | Rt+TMZ | 12.0 | P/O | 2.8 | none | No |
|  | 33 | 66, F | GBM | STR | Rt+TMZ+BCNU | 15.0 | F | 2.5 | none | No |
|  | 34 | 56, M | GBM | GTR | Rt+HU+PCV | 9.0 | T | 10.93 | TMZ+TAM+IRN+^125^I | No |
|  | 35 | 49, M | GBM | GTR | Rt+TMZ+SRS+BCNU+  isoretinoin | 16.0 | F | 4.9 | CBP | No |
|  | 36 | 47, F | GBM | GTR | Rt+SRS+TAM+^125^I | 13.5 | T | 8.5 | ^125^I | No |
|  | 37 | 56, M | GBM | STR | Rt+TMZ+SRS | 18.0 | T | 4.8 | BCNU+IRN+CBP | No |
|  | 38 | 72, F | GBM | GTR | Rt+Chemo# | 11.0 | T | 15.6 | R125177 | No |
|  | 39 | 41, M | AA, GBM | GTR | SRS+TAM+PAA+CBP | 26.0 | P/O | 20.9 | Rt+VP-16+^125^I | No |

(*continued*)

Supplementary table 1: Summary all the cases of IEBCs (*continued*)

| Study | Case | Age (yrs) | Initial diagnosis | EOR of primary glioma | Adjuvant therapy for primary glioma | Time to SGS (months) | Location of SGS | OS from SGS (months) | Therapy for SGS | Extracranial metastasis |
| --- | --- | --- | --- | --- | --- | --- | --- | --- | --- | --- |
| 2010(11) | 40 | 47, M | GBM | GTR | Rt+SRS | 16.0 | P | 17.3 | ^125^I | No |
|  | 41 | 53, F | GBM | GTR | Rt+TMZ+VP-16 | 25.0 | T | 10.3 | BCNU+CBP | No |
|  | 42 | 51, F | GBM | STR | none | 0.5 | T | 46.4 | Rt+TMZ+SRS | No |
|  | 43 | 61, F | GBM | STR | Rt+TMZ | NR | T | 4.6 | None | No |
|  | 44 | 57, M | GBM | GTR | Rt+TMZ | 3.0 | F/T | NA | Thalidomide+BCNU | No |
|  | 45 | 45, F | GBM | GTR | Rt+TMZ+IRN+BCNU+ flutamide+Gliadel wafer | 12.0 | T/P | NA | TMZ+E | No |
|  | 46 | 45, M | LGA, GBM | GTR | Rt+PCV+TMZ | 65.0 | T | NA | IRN+TAM | No |
| 2011(12) | 47 | 48, M | GBM | GTR | Rt+TMZ | 17.0 | T | 5.0 | Bev+IRN | No |
| 2011(13) | 48 | 48, M, | GBM | GTR | Rt+TMZ | 23.0 | T | 4.0 | NR | No |
| 2011(14) | 49 | 43, F | AA | STR | Rt+TMZ | 50.0 | T | 1.0 | none | No |
| 2011(15) | 50 | 62, M | GBM | STR | Rt+TMZ | 18.0 | PO | NR | NR | No |
| 2012(16) | 51 | 41, M | AO | GTR | TMZ | 60.0 | F | NR | Rt+TMZ | No |
| 2013(17) | 52 | 57, F | GBM | GTR | Rt+TMZ | 57.0 | scalp | 6.5 | Rt+MAI | Yes |
| 2013(18) | 53 | 57, M | LGO | STR | Rt | 15.0 | Cc | 6.0 | TMZ | No |
| 2014(19) | 54 | 4, M | LGA | PR | CBP+ VCR | 47.0 | T | 84* | Rt+TMZ、 | No |
| 2014(20) | 55 | 61, M | LGA | NR | none | 36.0 | NR | NR | NR | NR |
| 2014(21) | 56 | 46, F | LOA | STR | none | 48.0 | F | 15* | Rt+TMZ+Bev | No |
| 2014(22) | 57 | 64, M | GBM | GTR | Rt+TMZ | 9.0 | F | 14.0 | SRS+TMZ+Bev+IRN | Yes |
| 2015(23) | 58 | 63, M | GBM | STR | Rt+TMZ | 14.0 | T | 2.0 | HCQ+ Vor | NR |
|  | 59 | 65, M | GBM | STR | Rt+TMZ+Bev | 17.0 | TP | 9.0 | Rt+Bev | NR |
|  | 60 | 66, M | GBM | STR | Rt+TMZ | 13.0 | FT | 12.0 | TMZ+ Gliadel wafer | NR |
|  | 61 | 60, M | GBM | GTR | Rt+TMZ+T and S + temsirolimus and erlotinib+LOM+Bev | 23.0 | TP | 9.0 | none | NR |
|  | 62 | 55, M | GBM | STR | Rt+TMZ+Gliadel wafer | 12.0 | TO | 3.0 | XL-184 | NR |
|  | 63 | 55, M | GBM | GTR | Rt+TMZ+Accutane+ Celebrex | 10.0 | T | 7.0 | Bev | NR |
|  | 64 | 60, M | GBM | GTR | Rt+TMZ | 9.0 | P | 5.0 | Bev+CBP | NR |
|  | 65 | 51, M | GBM | GTR | Rt+TMZ+ CBP+ Gefitinib | 9.0 | T | 10.0 | Rt+TMZ+IRN+ sarasar | NR |
|  | 66 | 68, M | GBM | GTR | Rt+TMZ+Accutane | 28.0 | P | 21.0 | CBP+EP+Bev | NR |
|  | 67 | 57, F | GBM | STR | Rt+TMZ+dasatinib+ adenovirus | 51.0 | PO | 2.0 | Rt+TMZ | NR |
| 2015(24) | 68 | 49, M | AA | NR | Rt+TMZ | 10.0 | T | NR | NR | No |
| 2016(25) | 69 | 60, F | GBM | STR | Rt+TMZ | 18.0 | T | 10.0 | Bev+CBP | Yes |
| 2016(26) | 70 | 63, M | GBM | GTR | Rt | 12.0 | F | 15.0 | Rt+TMZ | Yes |
| 2016(27) | 71 | 56, M | GBM | GTR | Rt+TMZ+carmustine+IRN+Taxol | 32.0 | Pt | 2.0 | none | Yes |
| 2017(28) | 72 | 57, M | GBM | GTR | Rt+TMZ+vaccination | 12.0 | subdural | 4.0 | SR | No |
| 2017(29) | 73 | 59, F | GG | Biopsy | none | 36.0 | P+Cing | NR | Rt+TMZ | No |
| 2018(30) | 74 | 53, M | LGO | GTR | PAV | 132.0 | F | 6* | Rt+TMZ | No |
| 2018(31) | 75 | 36, M | LGO | STR | Rt+PCV | 72.0 | F | 6* | TMZ | No |

(*continued*)

Supplementary table 1: Summary all the cases of IEBCs (*continued*)

| Study | Case | Age (yrs) | Initial diagnosis | EOR of primary glioma | Adjuvant therapy for primary glioma | Time to SGS (months) | Location of SGS | OS from SGS (months) | Therapy for SGS | Extracranial metastasis |
| --- | --- | --- | --- | --- | --- | --- | --- | --- | --- | --- |
| 2019(32) | 76 | 50-59, M | 0 | STR | Rt+TMZ | 13.2 | F | 9.0 | SR | No |
|  | 77 | 60-69, F | GBM | GTR | Rt+TMZ | 10.2 | P | 8.0 | Bev+CCNU+SR | No |
|  | 78 | 60-69, M | GBM | STR | Rt+TMZ | 5.4 | F | 6.6 | Bev+IRN+Rt | No |
|  | 79 | 50-59, M | GBM | Biopsy | Rt+TMZ | 19.3 | F/P | 1.2 | NONE | NR |
|  | 80 | 50-59, M | GBM | STR | Rt+TMZ | 5.7 | F/P | 6.7 | Bev+IRN | No |
|  | 81 | 60-69, F | GBM | STR | Rt+TMZ | 10.9 | T | 7.7 | Bev+IRN+SR | No |
|  | 82 | 20-29, F | GBM | GTR | Rt+TMZ+IRN+Bev+  Ce+Tor | 19.6 | P | 2.2 | Cilengetide | No |
| 2019(33) | 83 | 74 | GBM | GTR | Rt+TMZ | 5.0 | subdural | 5.5* | SR | No |
| 2019(34) | 84 | 67 | GBM | GTR | Rt+TMZ | 6.5 | T | 18.5 | Nivolumab+  Bev | No |
| 2020(35) | 85 | 53 | GBM | GTR | Rt+TMZ+Bev+ Gliadel wafer | 15.0 | T/P | 5.0 | NR | No |
| 2020(36) | 86 | 12 | GBM | NR | Rt+Chemo# | 8.0 | dural | 5.0 | Rt+Chemo# | Yes |
| 2021(37) | 87 | 59 | GBM | STR | Rt+TMZ+Bev+CBP+ olaparib | 10.0 | basal ganglia | 1.0 | Bev | No |
| 2021(38) | 88 | 81 | GBM | STR | Rt+TMZ+SRS | 21.0 | T | 6.0* | NR | No |
| 2021(39) | 89 | 59 | GBM | GTR | Rt+TMZ+Bev | 10.0 | F/P | 5.0 | NR | No |
| Our cases | 90 | 54, F | GBM | GTR | SRS+TMZ | 10.5 | T | 8.5 | TMZ | No |
|  | 91 | 48, M | GBM | GTR | SRS+TMZ | 6.8 | T | 5.3 | none | No |
|  | 92 | 42, F | AO, GBM | GTR | TMZ | 21.3 | F | 7.5 | TMZ | No |
|  | 93 | 49, M | GBM | GTR | RT+TMZ | 14.6 | T | 3.1 | none | No |
|  | 94 | 41, F | GBM | GTR | RT+TMZ | 11 | F | 16.7 | SR+Bev | No |
|  | 95 | 46, M | GBM | GTR | RT+TMZ | 13.8 | T | 4.7 | TMZ | No |
|  | 96 | 49, M | GBM | GTR | SRS | 13.6 | T | 3.3 | none | No |
|  | 97 | 38, M | AO | GTR | none | 11.3 | T/I | 2.1 | none | No |
|  | 98 | 59, F | GBM | PR | none | 3.9 | F/T | 1.5 | none | No |
|  | 99 | 50, F | AA | GTR | Rt | 19.7 | T | 7.3 | TMZ | No |
|  | 100 | 21, M | AA | GTR | SRS+TMZ | 29.1 | Thoracic/  lumbar | 2 | none | Yes |
|  | 101 | 67, M | GBM | GTR | RT+TMZ | 19.8 | T/P | 25.5 | TMZ | No |
|  | 102 | 45, M | GBM | GTR | RT+TMZ | 12.5 | F | 1.5 | none | No |
|  | 103 | 63, M | GBM | GTR | RT+TMZ | 14.6 | T | 4.4 | none | No |
|  | 104 | 45, F | GBM | GTR | RT+TMZ | 40.2 | T | 11.5* | TMZ | No |
|  | 105 | 40, F | AA | GTR | RT+TMZ | 16 | F | 6.1 | TMZ | No |
|  | 106 | 27, F | LGO | GTR | None | 62.3 | T/I, | 16.3 | Rt+TMZ | No |
|  | 107 | 58, M | GBM | GTR | RT+TMZ | 62.4 | T | 2.8 | none | No |

M, male; F, female; GBM, glioblastoma; SGS, secondary gliosarcoma; AE, anaplastic ependymoma; LGO, low grade oligodendroglioma; AO, anaplastic oligodendroglioma; LGA, low grade astrocytoma; AA, anaplastic astrocytoma; LOA, low grade oligoastrocytoma; PA, pilocytic astrocytoma; GG, ganglioglioma; EOR, extent of resection; GTR, gross total resection; STR, subtotal resection; PR, partial resection; NR, not reported; B, brainstem; P, parietal lobe; O, occipital lobe; F, frontal lobe; T, temporal lobe; I, insula; Cb, cerebellum; Cc, corpus callosum; Pt, pterygomaxillary; Cing, cingulate gyrus; Rt radiation therapy; Chemo, chemotherapy; SRS, stereotactic radiosurgery; PCV, procarbazine, CCNU, and vincristine; TMZ, temozolomide; CIS, cisplatin; DOX, doxorubicin; SR, surgical resection; HU, hydroxyurea; CBP, carboplatin; E, erlotinib; TAM, tamoxifen; Paz, pazopanib; PAA, phosphonoacetic acid; VP-16, etoposide; IRN, irinotecan; IL-13, IL-13 pseudomonal toxin; VCR, vincristine; MAI, mesna, adriamycin and ifosfamide; T, tipifarnib; S, sorafenib; PAV, procarbazine, ACNU and vincristine; HCQ, hydroxychloroquine; Vor, vorinostat; EP, etoposide; Ce, cetuximab; Tor, torisel;

# The chemotherapy regimen was unavailable.

*The patient remains alive in the literature reports or at the end of follow-up.

**REFERENCES**

1. Reis RM, Könü-Lebleblicioglu D, Lopes JM, Kleihues P, Ohgaki H. Genetic profile of gliosarcomas. *The American journal of pathology* (2000) 156(2):425-32. Epub 2000/02/10. doi: 10.1016/s0002-9440(10)64746-3. PubMed PMID: 10666371; PubMed Central PMCID: PMCPMC1850048.

2. Lieberman KA, Fuller CE, Caruso RD, Schelper RL. Postradiation gliosarcoma with osteosarcomatous components. *Neuroradiology* (2001) 43(7):555-8. Epub 2001/08/22. doi: 10.1007/s002340000531. PubMed PMID: 11512585.

3. Behling E, Birbe R, Veznadaroglu E, Andrews DW, Flanders A, Kenyon LC. Gliosarcoma arising from an anaplastic ependymoma: A case report of a rare entity. *Human Pathology* (2004) 35(4):512-6. doi: 10.1016/j.humpath.2003.10.018.

4. Deb P, Sharma MC, Chander B, Mahapatra AK, Sarkar C. Giant cell glioblastoma multiforme: report of a case with prolonged survival and transformation to gliosarcoma. *Child's nervous system : ChNS : official journal of the International Society for Pediatric Neurosurgery* (2006) 22(3):314-9. Epub 2005/09/01. doi: 10.1007/s00381-005-1239-9. PubMed PMID: 16133270.

5. Rodriguez FJ, Scheithauer BW, Jenkins R, Burger PC, Rudzinskiy P, Vlodavsky E, et al. Gliosarcoma arising in oligodendroglial tumors ("oligosarcoma"): a clinicopathologic study. *Am J Surg Pathol* (2007) 31(3):351-62. Epub 2007/02/28. doi: 10.1097/01.pas.0000213378.94547.ae. PubMed PMID: 17325476.

6. Jager B, Schuhmann MU, Schober R, Kortmann RD, Meixensberger J. Induction of gliosarcoma and atypical meningioma 13 years after radiotherapy of residual pilocytic astrocytoma in childhood. *Pediatr Neurosurg* (2008) 44(2):153-8. Epub 2008/01/31. doi: 10.1159/000113120. PubMed PMID: 18230932.

7. Frank S, Cordier D, Tolnay M, Rosenblum MK. A 28-year-old man with headache, visual and aphasic speech disturbances. *Brain pathology (Zurich, Switzerland)* (2009) 19(1):163-6. Epub 2008/12/17. doi: 10.1111/j.1750-3639.2008.00241.x. PubMed PMID: 19076784; PubMed Central PMCID: PMCPMC8094774.

8. Kano T, Ikota H, Wada H, Iwasa S, Kurosaki S. A case of an anaplastic ependymoma with gliosarcomatous components. *Brain Tumor Pathol* (2009) 26(1):11-7. Epub 2009/05/02. doi: 10.1007/s10014-008-0240-x. PubMed PMID: 19408092.

9. Bekar A, Kahveci R, Tolunay S, Kahraman A, Kuytu T. Metastatic gliosarcoma mass extension to a donor fascia lata graft harvest site by tumor cell contamination. *World Neurosurg* (2010) 73(6):719-21. Epub 2010/10/12. doi: 10.1016/j.wneu.2010.03.015. PubMed PMID: 20934163.

10. Cheong JH, Kim CH, Kim JM, Oh YH. Transformation of intracranial anaplastic astrocytoma associated with neurofibromatosis type I into gliosarcoma: case report. *Clin Neurol Neurosurg* (2010) 112(8):701-6. Epub 2010/05/15. doi: 10.1016/j.clineuro.2010.04.012. PubMed PMID: 20466481.

11. Han SJ, Yang I, Otero JJ, Ahn BJ, Tihan T, McDermott MW, et al. Secondary gliosarcoma after diagnosis of glioblastoma: Clinical experience with 30 consecutive patients. *Journal of Neurosurgery* (2010) 112(5):990-6. doi: 10.3171/2009.9.JNS09931.

12. Andaloussi-Saghir K, Oukabli M, el Marjany M, Sifat H, Hadadi K, Mansouri H. Secondary gliosarcoma after the treatment of primary glioblastoma multiforme. *North American Journal of Medical Sciences* (2011) 3(11):527-30. doi: 10.4297/najms.2011.3527.

13. Pimentel J, Marques J, Pereira P, Roque L, Martins C, Campos A. Sarcoma with true epithelial differentiation secondary to irradiated glioblastoma. *Clin Neuropathol* (2011) 30(6):307-12. Epub 2011/10/21. doi: 10.5414/np300390. PubMed PMID: 22011736.

14. Romeike BF, Chen Y, Walter J, Petersen I. Diagnostic utility of IDH1- and p53-mutation analysis in secondary gliosarcoma. *Clin Neuropathol* (2011) 30(5):231-4. Epub 2011/10/01. doi: 10.5414/np300375. PubMed PMID: 21955926.

15. Shivane A, Fewings P. Pathologic features of an unusual case of secondary gliosarcoma. *Neuropathology and Applied Neurobiology* (2011) 37:40-1.

16. Vajtai I, Vassella E, Hewer E, Kappeler A, Reinert MM. Sarcomatous evolution of oligodendroglioma ("oligosarcoma"): confirmatory report of an uncommon pattern of malignant progression in oligodendroglial tumors. *Pathology, research and practice* (2012) 208(12):750-5. Epub 2012/10/30. doi: 10.1016/j.prp.2012.09.009. PubMed PMID: 23102810.

17. Dawar R, Fabiano AJ, Qiu J, Khushalani NI. Secondary gliosarcoma with extra-cranial metastases: a report and review of the literature. *Clin Neurol Neurosurg* (2013) 115(4):375-80. Epub 2012/07/17. doi: 10.1016/j.clineuro.2012.06.017. PubMed PMID: 22795300.

18. Hiniker A, Hagenkord JM, Powers MP, Aghi MK, Prados MD, Perry A. Gliosarcoma arising from an oligodendroglioma (oligosarcoma). *Clin Neuropathol* (2013) 32(3):165-70. Epub 2012/12/21. doi: 10.5414/np300577. PubMed PMID: 23254140.

19. Burzynski SR, Janicki TJ, Burzynski GS, Marszalek A. Long-term survival (>13 years) in a child with recurrent diffuse pontine gliosarcoma: a case report. *J Pediatr Hematol Oncol* (2014) 36(7):e433-9. Epub 2013/10/19. doi: 10.1097/mph.0000000000000020. PubMed PMID: 24136026; PubMed Central PMCID: PMCPMC4217198 member of the Board of Directors of BRI Inc.T.J.J. is the Vice President of Clinical Trials at BRI Inc. All authors are employed by Burzynski Clinic.

20. Codispoti KE, Mosier S, Ramsey R, Lin MT, Rodriguez FJ. Genetic and pathologic evolution of early secondary gliosarcoma. *Brain Tumor Pathol* (2014) 31(1):40-6. Epub 2013/01/18. doi: 10.1007/s10014-012-0132-y. PubMed PMID: 23324827; PubMed Central PMCID: PMCPMC3991122.

21. Rech F, Rigau V, Fabbro M, Kerr C, Gauchotte G, Taillandier L, et al. A nonradiated grade II glioma that underwent delayed malignant transformation to a gliosarcoma with meningeal growth and dissemination. *Journal of neurological surgery Part A, Central European neurosurgery* (2014) 75(6):485-90. Epub 2014/06/28. doi: 10.1055/s-0034-1372437. PubMed PMID: 24971682.

22. Schindler G, Capper D, Korshunov A, Schmieder K, Brenke C. Spinal metastasis of gliosarcoma: array-based comparative genomic hybridization for confirmation of metastatic spread. *Journal of clinical neuroscience : official journal of the Neurosurgical Society of Australasia* (2014) 21(11):1945-50. Epub 2014/07/30. doi: 10.1016/j.jocn.2014.03.034. PubMed PMID: 25065849.

23. Cachia D, Kamiya-Matsuoka C, Mandel JJ, Olar A, Cykowski MD, Armstrong TS, et al. Primary and secondary gliosarcomas: clinical, molecular and survival characteristics. *J Neurooncol* (2015) 125(2):401-10. Epub 2015/09/12. doi: 10.1007/s11060-015-1930-y. PubMed PMID: 26354773; PubMed Central PMCID: PMCPMC8323586.

24. Niu H, Wang K, Song Z, Sun W. Secondary gliosarcoma arising from an anaplastic astrocytoma: A case report and review of the literature. *Neurosurgery Quarterly* (2015) 25(2):271-4. doi: 10.1097/WNQ.0000000000000042.

25. Calderon-Garcidueñas AL, Idbaih A, Galanaud D, Duyckaerts C, Bielle F. Dural and osteolytic sarcomatoid relapse of a secondary gliosarcoma with tryptase immunoreactivity. *Clin Neuropathol* (2016) 35(3):154-8. Epub 2016/01/20. doi: 10.5414/np300916. PubMed PMID: 26784965.

26. Choi TM, Cheon YJ, Jung TY, Lee KH. A Stable Secondary Gliosarcoma with Extensive Systemic Metastases: A Case Report. *Brain tumor research and treatment* (2016) 4(2):133-7. Epub 2016/11/22. doi: 10.14791/btrt.2016.4.2.133. PubMed PMID: 27867925; PubMed Central PMCID: PMCPMC5114185.

27. Mason A, Villavicencio AT, Nelson EL, Forsythe RC, Burneikiene S. Post-Treatment Gliosarcoma Extension into the Pterygomaxillary Fossa: Literature Review and Case Report. *Cureus* (2016) 8(7):e700. Epub 2016/10/05. doi: 10.7759/cureus.700. PubMed PMID: 27699141; PubMed Central PMCID: PMCPMC5040629.

28. Meyer RM, Miller CA, Coughlin DJ, Rymarczuk G, Szuflita NS, Cirivello MJ, et al. Glioblastoma recurrence, progression, and dissemination as a purely subdural gliosarcoma. *J Neurooncol* (2017) 132(3):521-2. Epub 2017/03/16. doi: 10.1007/s11060-017-2397-9. PubMed PMID: 28290000.

29. Qiu L, Tang YL, King NK, Chuah KL, Lai SH, Lim KH, et al. IDH1 mutant negative ganglioglioma progression to gliosarcoma. *Interdisciplinary Neurosurgery: Advanced Techniques and Case Management* (2017) 9:41-4. doi: 10.1016/j.inat.2017.02.005.

30. Tanaka S, Hitotsumatsu T, Sugita Y, Ishido K, Ito O, Hatae R, et al. Gliosarcoma arising from oligodendroglioma (Oligosarcoma): A case report with genetic analyses. *Pathol Int* (2018) 68(10):567-73. Epub 2018/09/25. doi: 10.1111/pin.12723. PubMed PMID: 30246385.

31. Yasuda T, Nitta M, Komori T, Kobayashi T, Masui K, Maruyama T, et al. Gliosarcoma arising from oligodendroglioma, IDH mutant and 1p/19q codeleted. *Neuropathology* (2018) 38(1):41-6. Epub 2017/08/16. doi: 10.1111/neup.12406. PubMed PMID: 28812310.

32. Frandsen S, Broholm H, Larsen VA, Grunnet K, Møller S, Poulsen HS, et al. Clinical Characteristics of Gliosarcoma and Outcomes From Standardized Treatment Relative to Conventional Glioblastoma. *Front Oncol* (2019) 9:1425. Epub 2020/01/11. doi: 10.3389/fonc.2019.01425. PubMed PMID: 31921679; PubMed Central PMCID: PMCPMC6928109.

33. Khanna R, Brahimaj B, Tchalukov K, Byrne K, Adogwa O, Jhaveri M, et al. A case of recurrent gliosarcoma mimicking subdural hematoma. *Interdisciplinary Neurosurgery: Advanced Techniques and Case Management* (2019) 16:3-6. doi: 10.1016/j.inat.2018.10.014.

34. Restrepo P, Yong R, Laface I, Tsankova N, Gnjatic S, Hormigo A, et al. Mapping tumoral and immune heterogeneity in PD-1 responsive glioblastoma. *Cancer Research* (2019) 79(13). doi: 10.1158/1538-7445.SABCS18-2528.

35. Hsu BH, Lee WH, Yang ST, Han CT, Tseng YY. Spinal metastasis of glioblastoma multiforme before gliosarcomatous transformation: a case report. *BMC Neurol* (2020) 20(1):178. Epub 2020/05/13. doi: 10.1186/s12883-020-01768-3. PubMed PMID: 32393192; PubMed Central PMCID: PMCPMC7216360.

36. Jeng F, Reynolds A. Retrobulbar chlorpromazine injection in a child with gliosarcoma invasion into the orbits. *BMJ Case Rep* (2020) 13(6). Epub 2020/06/12. doi: 10.1136/bcr-2019-233394. PubMed PMID: 32522719; PubMed Central PMCID: PMCPMC7287501.

37. Kiang KM, Chan AA, Leung GK. Secondary gliosarcoma: the clinicopathological features and the development of a patient-derived xenograft model of gliosarcoma. *BMC Cancer* (2021) 21(1):265. Epub 2021/03/13. doi: 10.1186/s12885-021-08008-y. PubMed PMID: 33706745; PubMed Central PMCID: PMCPMC7948380.

38. Kim DW, Moon HC, Kim YG, Dho YS. The role of gamma knife radiosurgery in occurrence of secondary gliosarcoma after treatment of glioblastoma: A case report. *Interdisciplinary Neurosurgery: Advanced Techniques and Case Management* (2021) 24. doi: 10.1016/j.inat.2020.101057.

39. Li J, Zhao YH, Tian SF, Xu CS, Cai YX, Li K, et al. Genetic alteration and clonal evolution of primary glioblastoma into secondary gliosarcoma. *CNS neuroscience & therapeutics* (2021). Epub 2021/10/05. doi: 10.1111/cns.13740. PubMed PMID: 34605602.
